# Social support as a buffer in the relationship between perceived stress and PTSD among South African university students

**DOI:** 10.1177/20551029261476115

**Published:** 2026-08-21

**Authors:** Anita Padmanabhanunni, Tyrone B. Pretorius

**Affiliations:** 1 Department of Psychology, 56390 University of the Western Cape, Cape Town, South Africa; 2 Monash University, Melbourne, Australia

**Keywords:** moderation analysis, perceived stress, social support, PTSD

## Abstract

Mental health research has increasingly moved beyond linear risk models that view adversity as having uniform effects on psychological outcomes, toward identifying protective factors that shape responses to stress. This study examined social support as one such factor in the relationship between perceived stress and PTSD among South African university students. Participants (*n* = 491) completed the Perceived Stress Scale, the PTSD Checklist for DSM-5, and the Multidimensional Scale of Perceived Social Support. Moderation analysis found no significant direct effect of social support on PTSD. However, overall support, support from significant others, friends, and family, each moderated the relationship between perceived stress and PTSD. Specifically, the association between perceived stress and PTSD was stronger at low levels of social support and weaker at high levels. These findings highlight the buffering role of perceived social support and suggest that interventions strengthening support from multiple sources may reduce the impact of stress on PTSD symptoms.

## Introduction

Contemporary mental health research has increasingly emphasized the limitations of simple risk–outcome models in explaining variability in psychological adjustment following adversity (Mancini, 2019; Padmanabhanunni et al., 2022; Pretorius, 2020). In response, studies have moved beyond examining only direct associations between stress exposure and mental health outcomes and have increasingly focused on examining the mechanisms and conditions that shape these relationships (Zell and Stockus, 2025). Moderation analysis represents one such approach. Moderators are defined as “a third variable that affects the direction and/or strength of the relation between an independent variable or predictor variable and a dependent or criterion variable” (Baron and Kenny, 1986: 1176).

Moderation analysis is particularly important in trauma research, where individuals exposed to comparable levels of stress may nevertheless differ markedly in their vulnerability to post-traumatic stress symptoms (Padmanabhanunni and Wiid, 2022). This variability suggests that stress exposure alone is insufficient to explain psychological adjustment following adversity. Instead, the impact of stress depends on the availability of protective resources that shape how individuals appraise, cope with, and recover from stressful or traumatic experiences. Moderation analysis allows us to determine whether a protective resource alters the strength or direction of the relationship between stress and psychological outcomes (Pretorius, 2020).

Prior studies have used moderation frameworks to demonstrate that protective resources do not merely exert a direct association with mental health outcomes, but may alter the strength of the relationship between stress and psychological distress. For instance, studies examining HIV-related stigma and mental health have used moderation analysis to show that social support may function as a stress-buffering resource, altering the extent to which stigma is associated with psychological distress (Brener et al., 2020; Lo Hog Tian et al., 2025). Similarly, adolescent mental health research has employed moderation models to demonstrate that parental support can attenuate the relationship between stress exposure and emotional difficulties (DeSmet et al., 2021; Qian et al., 2024). Furthermore, studies conducted during the COVID-19 pandemic illustrate the value of moderation analysis, demonstrating that protective factors such as resilience, sense of coherence, and psychological flexibility can shape the strength of the association between pandemic-related stressors and mental health outcomes among diverse populations (Havnen et al., 2020; Pakenham et al., 2020; Paterson et al., 2024). Collectively, these studies demonstrate that moderation modelling is useful for moving beyond direct-effect explanations by identifying the protective resources that may buffer vulnerability under conditions of elevated stress.

Social support has been conceptualized as a potentially protective resource for mental health and psychological adjustment (Tindle et al., 2022; Xu et al., 2025; Zell and Stockus, 2025). The current study, therefore, focused on the role of social support in the relationship between perceived stress and post-traumatic stress disorder (PTSD) among students attending a historically disadvantaged institution (HDI) in South Africa. Perceived stress refers to the degree to which individuals appraise their lives as unpredictable, uncontrollable, and overwhelming (Cohen et al., 1983). This conceptualization of perceived stress is aligned with the transactional model of stress and coping (Lazarus and Folkman, 1984), which emphasizes that the psychological impact of stressors depends on how these demands are appraised relative to available coping resources. Accordingly, the findings of the present study should be interpreted as reflecting the association between subjective stress appraisal and PTSD symptoms. PTSD is a mental health disorder that arises following exposure to potentially traumatic events. It is characterized by four broad symptom categories, namely, intrusive re-experiencing, avoidance of reminders of the traumatic event, negative alterations in cognition and mood, and heightened physiological arousal and reactivity (American Psychiatric Association, 2022). For university students, PTSD may impair academic performance and interfere with social relationships and engagement with university life. These consequences are particularly important in HDI settings, where students may already be navigating academic demands, financial strain, family expectations, and structural inequalities (Padmanabhanunni, 2020; Woldegiorgis and Chiramba, 2024).

HDIs refer to universities in South Africa that were created under apartheid as part of a racially segregated and unequal higher education system. Although significant institutional changes have occurred since the advent of democracy, these universities continue to serve student populations that are predominantly drawn from working-class, rural, and socio-economically disadvantaged communities (Badat, 2023; Padmanabhanunni and Wiid, 2022). For these students, university study frequently involves more than individual academic advancement. Instead, it is closely tied to family aspirations, intergenerational mobility, and the possibility of altering the socio-economic trajectory of households and communities (Webb, 2021; Woldegiorgis and Chiramba, 2024). Social support in this context may therefore assume a distinctive meaning. It may include encouragement but it may also be complicated by financial strain and the pressure students experience to succeed on behalf of others (Fongwa, 2021; Webb, 2021).

In contemporary research, social support is generally understood as the interpersonal resources individuals access through their social relationships, which may buffer the impact of stressors and facilitate coping during periods of adversity (Zell and Stockus, 2025). The construct has been conceptualized in several ways. One approach focuses on the type or function of support provided, including emotional, instrumental, informational, and appraisal support (Langford et al., 1997; Shumaker and Brownell, 1984).

A second approach examines the source of support, commonly distinguishing between support derived from family members, friends, or significant others (Zimet et al., 1988). The literature further distinguishes between received or enacted support and perceived social support. Received support refers to the actual supportive behaviors or assistance provided to an individual, whereas perceived support reflects the individual’s belief that support is available, accessible, and adequate when needed (Barrera Jr, 1986; Shumaker and Brownell, 1984). Perceptions of being supported have consistently been identified as beneficial in promoting a sense of security, belonging, and coping efficacy, even in the absence of direct supportive action (Jakobsen et al., 2022). Accordingly, perceived social support is frequently regarded as a key psychosocial resource that may mitigate psychological distress and protect against adverse mental health outcomes (Berglund et al., 2025; Jakobsen et al., 2022; Liao et al., 2022).

Existing South African studies have highlighted high levels of trauma exposure and PTSD among university students (Bantjes et al., 2023; Coetzee et al., 2024), with one study reporting that 97.6% of students sampled had experienced at least one traumatic event in their lifetime (Padmanabhanunni, 2020). In a context where trauma exposure is widespread, social support may also assume a qualitatively different meaning from that implied in conventional individual-level models of coping. For students attending an HDI, support may also reflect shared experience, mutual recognition, and collective meaning-making among peers and families who have faced comparable adversities. In this context, perceived social support may provide validation, reduce feelings of isolation, and strengthen a sense of belonging among students whose distress is shaped by both individual trauma histories and broader structural inequalities (Motsabi et al., 2020). Understanding the protective role of social support, therefore, remains relevant among student populations where exposure to trauma and ongoing stressors may be widespread.

The current study was informed by the transactional model of stress and coping, which conceptualizes stress as a function of individuals’ appraisal of environmental demands relative to available coping resources (Lazarus and Folkman, 1984). Within this framework, perceived social support represents an interpersonal coping resource that may influence whether stress is experienced as overwhelming or manageable. This perspective is also consistent with the stress-buffering hypothesis, which proposes that social support may protect mental health through its general beneficial effects and by reducing the adverse psychological impact of stress under conditions of heightened strain. Moderation analysis was therefore selected because it provides a direct test of whether perceived social support changes the magnitude of the association between perceived stress and PTSD symptoms. This approach is analytically appropriate when the research question concerns conditional effects; that is, whether the relationship between a predictor and an outcome differs across levels of a third variable (Pretorius, 2020).

This differs from models examining only direct effects, which assume that perceived stress and social support contribute independently and additively to PTSD symptoms. It also differs from mediation analysis, which would imply that perceived stress influences PTSD symptoms through social support as an intervening mechanism. Given that the present study is concerned with the protective conditions under which perceived stress may be more or less strongly associated with PTSD, moderation provides the most theoretically aligned approach.

We hypothesized that higher perceived stress would be associated with greater PTSD symptoms. We further hypothesized that perceived social support would be directly associated with PTSD symptoms and would moderate the relationship between perceived stress and PTSD, such that the association would be weaker at higher levels of perceived social support.

## Materials and method

### Participants and procedure

The participants (*n* = 491) were students enrolled at a university in the Western Cape province of South Africa. The study took place between November 2023 and November 2024. Google Forms was used to develop an electronic questionnaire that consisted of the instruments described in the Measures section. The electronic link to this questionnaire, together with an invitation to participate in the study were emailed to a random sample of 4000 students. The survey landing page included the participant information sheet and consent form. This page provided information on the purpose of the study, the voluntary nature of participation, confidentiality and anonymity, potential risks and benefits, and the research team’s contact details. Participants were informed that they could decline to participate or withdraw before submitting their responses without penalty. To proceed to the questionnaire, participants were required to confirm that they had read and understood the information provided and that they consented to participate in the study.

A sensitivity analysis conducted with the package “pwr2ppl” (Aberson, 2019) in R (R Development Core Team, 2020) found that, assuming a small effect (*r* = 0.20) for the relationship between the interaction term (independent variable X moderator) and the dependent variable, a sample size of 385 would provide 80% power to detect a significant interaction, while the current sample of 491 provided 99.6% power at α = 0.05. We used a small effect size because a 30-year review of moderation effects found that a small ΔR^2^ of less than 1% is the norm in the field (Aguinis et al., 2005).

The majority of the sample were women (64.8%) who studied at the undergraduate level (92.5%). The mean age of the sample was 21.22 years (*SD* = 3.52). Most of the participants came from urban areas (64.8%). Participants were from all nine provinces in South Africa, but the largest concentration was drawn from the Western (32.8%) and Eastern (27.5%) Cape.

### Measures

Participants completed the following standardized questionnaires: the Perceived Stress Scale10 (PSS10: Cohen, 1988), the Multidimensional Scale of Perceived Social Support (MSPSS: Zimet et al., 1988), and the Post-Traumatic Stress Disorder Checklist for DSM-5 (PCL-5: Blevins et al., 2015).

The PSS10 is a 10-item measure of the extent to which people appraise events or situations in their life as stressful. The items of the PSS are scored on a 5-point scale ranging from 0 (*never*) to 4 (*very often*), and higher scores represent higher levels of perceived stress. An example of an item of the PSS is “How often have you felt that you were unable to control the important things in your life?” In the present study, the four positively worded items were reverse-scored, and a total perceived stress score was computed by summing responses across all 10 items, with possible scores ranging from 0 to 40. This scoring procedure is consistent with the original scoring approach for the PSS10. In the development study of the PSS10, Cohen (1988) reported an estimate of internal consistency of .75 for the scale in a large probability sample in the United States. A psychometric study of the PSS10 in a sample of university students in South Africa reported a McDonald’s omega of .89, reflecting high reliability of PSS scores (Pretorius, 2023).

The MSPSS is a 12-item measure of perceptions of social support from three sources: family (e.g., my family really tries to help me), friends (e.g., I can count on my friends when things go wrong), and a significant other (e.g., I have a special person who is a real source of comfort to me). The items of the MSPSS are scored on a 7-point scale ranging from 1 (*very strongly disagree*) to 7 (*very strongly agree*), and higher scores reflect perceptions of higher levels of social support. In addition to the three different sources of support, an overall total score is also derived. In the present study, mean scores were computed for the total MSPSS and for the family, friends, and significant other subscales, consistent with the scoring approach used in the original validation study. The author of the MSPSS reported Cronbach’s alphas of .88 for the total scale, as well as .91, .87, and .85 for the significant other, family, and friends subscales, respectively (Zimet et al., 1988). A South African study with a sample of university students reported Cronbach’s alphas and McDonald’s omegas of .93, .92, and .94 for the significant other, family, and friends subscales, respectively (Padmanabhanunni et al., 2023). 

The PCL-5 is a 20-item measure of the severity of PTSD symptoms. Participants respond to the 20 items using a 5-point scale that ranges from 0 (*not at all*) to 4 *(extremely*), and higher scores reflect higher levels of PTSD. An example of an item of the PCL-5 is “how much were you bothered by avoiding memories, thoughts, or feelings related to the stressful experience?” In the present study, a total PTSD symptom severity score was computed by summing responses across all 20 items, with possible scores ranging from 0 to 80. This is consistent with the standard scoring procedure for the PCL-5. In the development study, the authors of the scale reported an internal consistency estimate of .94 for the PCL-5. While the PCL-5 is often used as both a total measure of PTSD symptoms as well as a measure of 5 different symptom clusters aligned to the DSM-V, a South African study provided empirical support for viewing the PCL-5 as a unidimensional measure of PTSD symptom severity (Padmanabhanunni and Pretorius, 2024), but highlighted that this should not impact on the clinical and diagnostic use of the PCL-5.

### Ethical considerations

The Biomedical Science Research Ethics Committee of the University of the Western Cape (Ethics reference: BM23/10/8, August 2025) provided ethical approval, and the study was conducted in accordance with the Declaration of Helsinki. No identifying particulars were collected. Participation was voluntary, and no incentives were offered for participation.

### Data analysis

All questionnaire items were set to compulsory in Google Forms, preventing item-level missing data. However, this procedure only ensured that participants responded to each item and should not be interpreted as evidence of response quality. IBM SPSS for Windows version 31 (IBM Corp., Armonk, NY, USA) was used to obtain distribution indices (skewness and kurtosis), correlations between study variables (Pearson’s *r*), estimates of internal consistency (Cronbach’s alpha) for all scales used in the study, and to determine the proportion of participants that met the criteria for probable PTSD. A study in South Africa used ROC curve analysis and suggested that a PCL-5 cutoff score of 32 provided optimal sensitivity and specificity (Kagee et al., 2022). Skewness and kurtosis values ranging from −2 to +2 indicate that the variables are approximately normally distributed (Hair et al., 2021). With regard to reliability, α > .70 is considered acceptable for research purposes (DeVon et al., 2007).

The PROCESS macro in SPSS was used to conduct the moderation analysis. In the moderation analysis, an interaction term is created by multiplying the presumed moderator (social support) and the independent variable (perceived stress). Prior to creating the interaction term, all variables were mean-centered to avoid multicollinearity, and we further examined variance inflation factors (VIFs) to assess whether multicollinearity remained problematic after mean-centering. VIFs greater than 5 reflect high multicollinearity (Hair et al., 2010). The total social support score was not included in the regression analysis because it is a linear combination of the support from significant others, friends, and family subscales.

If the interaction term is significant, the nature of the interaction is examined by comparing the relationship between stress and PTSD at three levels of social support: 1 *SD* below the mean, at the mean, and 1 *SD* above the mean. The interaction is also visually presented using plots of simple slopes at these three levels.

## Results

Using a cutoff score of 32 for the PCL-5 resulted in 52.3% of the participants meeting the criteria for probable PTSD. As a result, associations between PTSD symptoms and other variables can be interpreted as reflecting variation across different levels of trauma-related distress.

Table 1 presents the intercorrelations, descriptive statistics, distribution indices for the study variables, and estimates of internal consistency for the scales used.

**Table 1.** Intercorrelations, descriptive statistics, distribution indices, and estimates of internal consistency.

Variable/Scale	1	2	3	4	5	6
1. Perceived stress	—	​	​	​	​	​
2. Social support	−.11*	—	​	​	​	​
3. Support from significant other	−.02	.89**	—	​	​	​
4. Support from family	−.19**	.83**	.60**	—	​	​
5. Support from friends	−.07	.87**	.69**	.56**	—	​
6. PTSD	.57**	−.08	−.04	−.15*	−.03	—
$\bar{X}$	21.70	58.01	20.06	18.93	19.01	32.57
*SD*	5.81	17.11	6.79	6.47	6.56	18.62
Skewness	0.45	−0.65	−0.70	−0.50	−0.63	0.12
Kurtosis	0.32	−0.32	−0.53	−0.67	−0.41	−0.61
α	.76	.93	.91	.88	.92	.94

*p < .05, **p < .001.

The indices of skewness (−0.70 to 0.45) and kurtosis (−0.67 to 0.32) in Table 1 were within an acceptable range, indicating that the data for all the variables were approximately normally distributed. The estimates of internal consistency ranged from .76 to .94, confirming that all the measurement instruments produced reliable scores.

Table 1 indicates that perceived stress was significantly negatively associated with overall social support (*r* = −.11, *p* = .02, small effect size) and support from family (*r* = −.19, *p* < .001, small effect size), and significantly positively associated with PTSD (*r* = .57, *p* < .001, large effect size). The obtained correlations would indicate that higher levels of perceived stress were associated with higher levels of PTSD, while higher levels of overall social support and support from family were associated with lower levels of perceived stress. Furthermore, support from family was significantly negatively associated with PTSD, indicating that higher levels of support from family were associated with lower levels of PTSD.

The VIFs ranged from 1.07 to 2.29, and all were below the recommended threshold of 5. Table 2 presents the direct and moderating effects resulting from the moderation analysis.

**Table 2.** Direct and moderating effects resulting from the moderation analysis.

Effect	B	SE	95% CI		β	p
			LL	UL		
Direct effects						
Social support → PTSD	−.04	.04	−.12	.04	−.04	.31
Support from significant others → PTSD	−.08	.10	−.28	.12	−.03	.42
Support from family → PTSD	−.12	.11	−.33	.09	−.04	.27
Support from friends → PTSD	.02	.11	−.19	.23	.01	.85
Perceived stress → PTSD	1.78	.12	1.54	2.01	.56	<.001
Moderating effects						
Stress X Social support → PTSD	−.02	.01	−.04	−.01	−.13	<.001
Stress X Support from significant others → PTSD	−.05	.02	−.08	−.01	−.10	.004
Stress X Support from family → PTSD	−.05	.02	−.09	−.02	−.11	.002
Stress X Support from friends → PTSD	−.05	.02	−.08	−.01	−.09	.009

**Figure 1.**
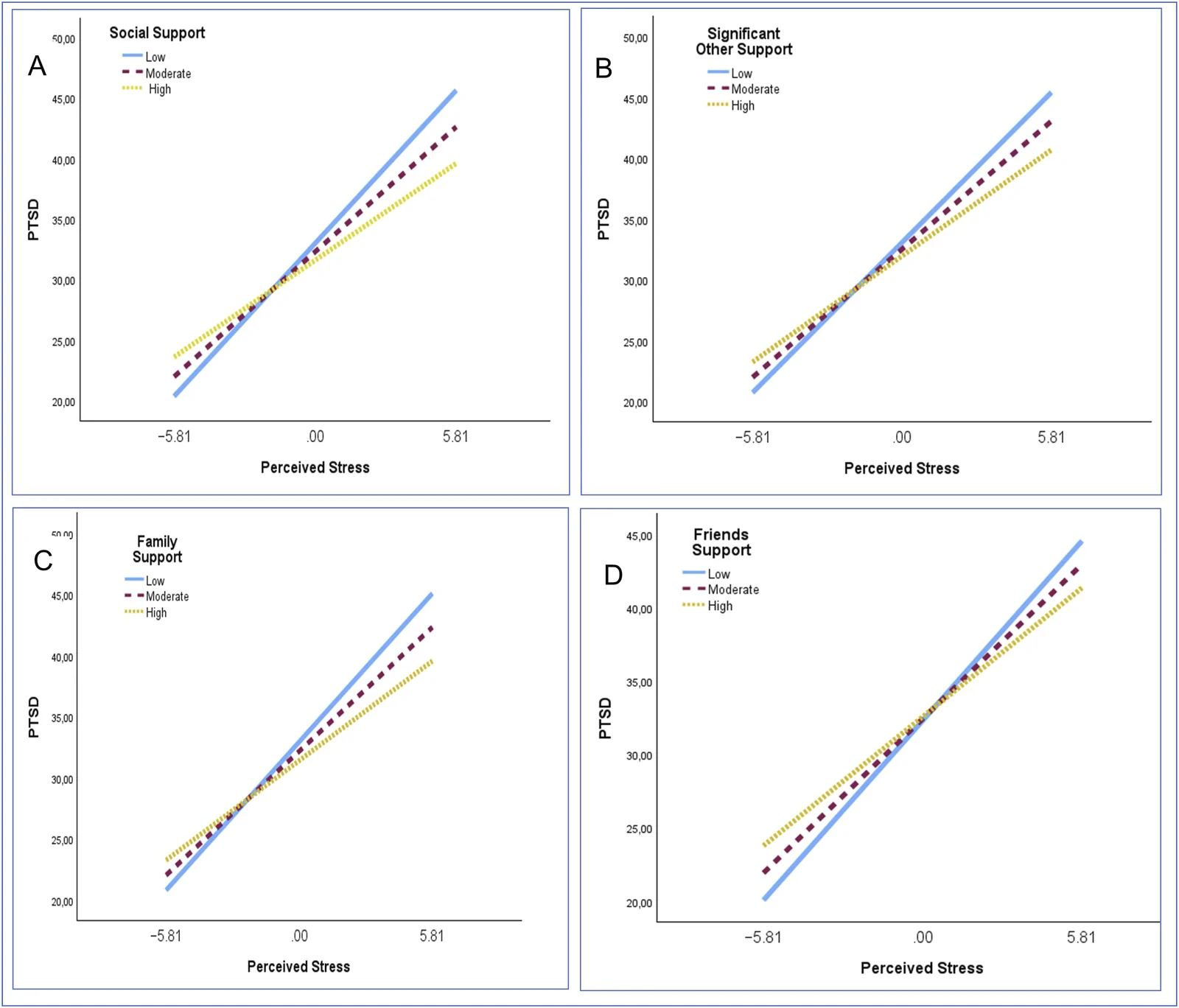
Plots of the relationship between perceived stress and PTSD at different levels of social support. *Note.* Figure 1(A) = the moderating role of social support, Figure 1(B) = the moderating role of support from significant other, Figure 1(C) = the moderating role of support from family, Figure 1(D) = the moderating role of support from friends.

Table 2 reflects that overall social support, nor any of the dimensions of social support had any significant direct effects on PTSD. On the other hand, the interaction between perceived stress and overall social support (β = −.13, *p* < .001), support from significant others (β = −.10, *p* = .004), support from family (β = −.11, *p* = .002), and support from friends (β = −.09, *p* = .009) were all significant, highlighting the moderating role of social support and its dimensions in the relationship between perceived stress and PTSD. The nature of the moderating effect is presented in Table 3, which highlights the relationship between perceived stress and PTSD at different levels of social support and its dimensions: 1 *SD* below the mean, at the mean, and 1 *SD* above the mean. The moderating effect is also demonstrated in Figure 1, which plots the relationship between perceived stress and PTSD for different levels of social support and its dimensions.

**Table 3.** The association between perceived stress and PTSD at different levels of social support and its dimensions.

Value of the moderator	B	SE	95% CI		β	*p*
			LL	UL		
Social Support						
1 *SD* below the mean	2.18	.16	1.87	2.49	.68	<.001
At the mean	1.78	.12	1.54	2.01	.56	<.001
1 SD above the mean	1.38	.17	1.04	1.72	.43	<.001
Support from significant others						
1 *SD* below the mean	2.13	.16	1.81	2.44	.66	<.001
At the mean	1.82	.12	1.58	2.05	.57	<.001
1 SD above the mean	1.50	.16	1.19	1.82	.47	<.001
Support from family						
1 *SD* below the mean	2.09	.15	1.80	2.39	.65	<.001
At the mean	1.75	.12	1.51	1.99	.55	<.001
1 SD above the mean	1.40	.17	1.06	1.75	.44	<.001
Support from friends						
1 *SD* below the mean	2.11	.16	1.79	2.42	.66	<.001
At the mean	1.81	.12	1.57	2.04	.57	<.001
1 SD above the mean	1.51	.17	1.18	1.84	.47	<.001

Table 3 indicates that the relationship between perceived stress and PTSD was stronger at low levels (1 *SD* below the mean) of overall support (β = .68), support from significant others (β = .66), support from family (β = .65), and support from friends (β = .66), than at high levels (1 *SD* above the mean) of overall support (β = .43), support from significant others (β = .47), support from family (β = .44), and support from friends (β = .47). The stronger relationship between perceived stress and PTSD at low levels of social support is further confirmed by the plots in Figure 1.

Figure 1 shows that the regression line of the relationship between perceived stress and PTSD was much steeper at low levels of social support, support from significant others, support from family, and support from friends than at high levels of social support and all its dimensions, pointing to the moderating role of social support.

**Figure 2.**
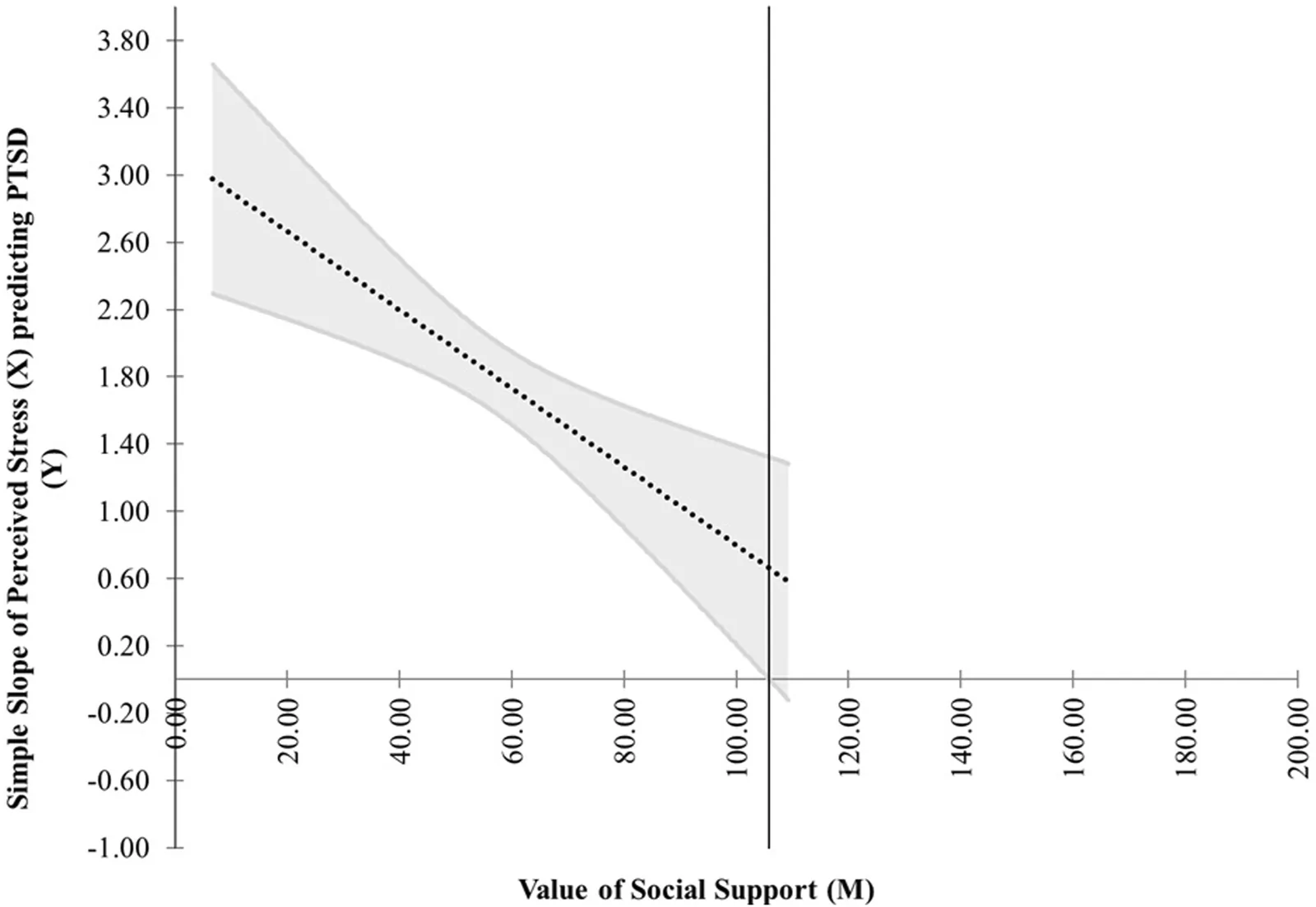
Johnson-Neyman plot identifying the social support score where the relationship between perceived stress and PTSD is no longer significant. *Note.* The vertical line indicates the value of the moderator where the relationship between perceived stress and PTSD is no longer significant.

The results of the Johnson-Neyman indicated there were no statistically significant transition points for all the moderators where the relationship between perceived stress and PTSD ceased to be significant, within the observed range of the moderators. The Johnson-Neyman plot for the total social support score is shown in Figure 2, as an example.

The plot identifies a social support score of 105.90 as the transition point for the relationship between perceived stress and PTSD, turning non-significant, whereas actual scores for social support can only range between 1 and 84. This indicates that, although the relationship between perceived stress and PTSD appears weaker at high levels of social support—as shown in Table 3—it remains statistically significant across the entire observed range of the moderator.

## Discussion

A growing body of mental health research has called for greater attention to the factors that shape differential psychological outcomes in the context of adversity (Heinz et al., 2025; Mancini, 2019). The current study investigated the role of social support in the relationship between perceived stress and PTSD among university students attending an HDI in South Africa. There were several significant findings.

First, the study confirmed that perceived stress was strongly associated with PTSD. This finding aligns with the existing literature in the field (Aslan and Çınar, 2023; Sun et al., 2025). However, it needs to be emphasized that PTSD is, by definition, anchored in exposure to a traumatic event. The severity and persistence of PTSD symptoms may be shaped by the extent to which individuals experience their current circumstances as stressful, uncontrollable, and overwhelming. In the present study, trauma exposure was not directly assessed. However, the strong association between perceived stress and PTSD suggests that trauma-related distress among students may be compounded by ongoing stressors in their academic, social, and material environments. Existing studies have highlighted that for students in HDIs, sources of stress can also include financial strain, food and housing insecurity, pressure to succeed, and concern about the well-being of families who may have made considerable sacrifices to support their studies (Themane and Mabasa, 2022; Woldegiorgis and Chiramba, 2024). As a result, perceived stress may reflect a complex combination of academic pressure, material hardship, family obligation, and the weight of collective expectations. However, these contextual stressors cannot be directly inferred from PSS scores. Instead, they provide an important backdrop for interpreting why perceptions of uncontrollability and overwhelm may be especially salient among students at an HDI.

Second, contrary to our hypothesis, social support did not show a significant direct effect on PTSD. Neither overall social support nor support from significant others, family, or friends had an effect on PTSD symptoms in the moderation models. This finding differs from the existing literature (Clapp and Gayle Beck, 2009; Johansen et al., 2022) and suggests that social support may not operate in a simple, linear manner in relation to trauma-related distress. In other words, having higher levels of perceived support may not, in itself, be sufficient to reduce PTSD symptoms in specific populations. One possible explanation for these differing findings may relate to the particular meaning of social support among students. For many students at HDIs, support from family and community may be accompanied by pressure to succeed, particularly where university education is viewed as a pathway to social mobility for the family. Thus, support may be experienced as a reminder of sacrifice and collective investment, and this may help explain the absence of a direct association with PTSD.

Third, social support moderated the relationship between perceived stress and PTSD. The relationship between perceived stress and PTSD was strongest when students reported low levels of overall support, support from significant others, support from family, and support from friends. Conversely, this relationship was weaker at higher levels of support. This pattern supports the stress-buffering model of social support, which proposes that supportive relationships may protect mental health by reducing the adverse psychological impact of stress (Johansen et al., 2022; Zell and Stockus, 2025). However, while social support significantly moderated the relationship between perceived stress and PTSD symptoms, this relationship remained statistically significant even at high levels of social support. This shows that although strong social support can help reduce distress, it is not enough to completely buffer the impact of perceived stress on PTSD. This is partly because PTSD includes components like cognitive (negative changes in thinking and intrusive re-experiencing) and neurobiological (hyper-arousal) symptoms, which cannot be managed solely through support from others.

The findings have several important implications for student mental health promotion in historically disadvantaged higher education contexts. Given the strong association between perceived stress and PTSD, universities should be attentive to the possibility that students presenting with academic difficulties, absenteeism, or disengagement may be experiencing trauma-related distress. Screening, early identification, referral pathways, and staff training may help institutions respond more appropriately to students whose academic functioning is affected by psychological distress (Miller et al., 2024).

The findings also suggest that social support should be understood as a key protective resource and highlight the need for universities to strengthen both formal and informal support systems. This can include broader relational and institutional strategies, such as peer mentoring, residence-based support, first-generation student programmes, and mechanisms that help students remain connected to supportive family and community networks. Such interventions may be especially important for students whose home environments are emotionally meaningful but materially constrained.

The finding that the relationship between perceived stress and PTSD was statistically significant at high levels of social support, despite social support’s moderating role, also has clinical implications. It indicates that social support alone may not be sufficient to fully counteract the psychological effects of perceived stress and should be considered along with other internal protective resources, such as psychological resilience and individual therapies like cognitive behavioral therapy.

The study had several limitations. The cross-sectional design precludes conclusions about causality or directionality. Although perceived stress was strongly associated with PTSD symptoms and social support moderated this relationship, it cannot be determined whether perceived stress contributed to PTSD symptoms, whether PTSD symptoms heightened students’ appraisal of stress, or whether these processes were reciprocal. The study relied on self-report measures, which may be affected by recall bias, response tendencies, and shared method variance. Although PTSD symptoms were assessed, the study did not directly assess trauma exposure, including the nature or severity of traumatic events. The absence of such data limits the extent to which the findings can explain the origins of students’ PTSD symptoms. The study assessed perceived social support rather than the quality, type, or actual availability of support. This is particularly relevant in an HDI context, where support may be emotionally meaningful but also associated with obligation, pressure, and material constraint.

The online survey did not include embedded attention-check items or other formal indicators of inattentive responding. Although the use of compulsory items prevented item-level missing data, forced responding does not preclude careless, random, or low-quality responses. Accordingly, the possibility that some responses may have reflected limited engagement with the questionnaire cannot be fully excluded. A further limitation concerns the operationalization of stress. The PSS measures perceived stress and, consequently, conclusions regarding the specific sources of stress experienced by students cannot be drawn from PSS scores alone. Future studies should include context-specific measures of academic, material, familial, and social stressors to provide a more precise account of the forms of stress that shape PTSD symptoms among students at historically disadvantaged institutions.

The finding that 47.7% of participants did not screen positive for probable PTSD while 52.3% did indicates that the sample is heterogeneous in PTSD symptom severity. However, in terms of generalizability, the findings may be most applicable to populations with similar trauma exposure, contextual stressors, or help-seeking characteristics. The results should therefore be generalized cautiously to lower-risk or general community/student populations. Furthermore, the study was conducted at a single historically disadvantaged university, and the findings may not be generalizable to students at other types of institutions or in different socio-economic and cultural contexts.

## Conclusion

The present study contributes to the literature by showing that perceived stress is strongly associated with PTSD among students attending an HDI in South Africa. Importantly, the study demonstrates that social support functions less as a direct predictor of PTSD and more as a moderator. Overall social support, as well as support from family, friends, and significant others, weakened the relationship between perceived stress and PTSD, suggesting that supportive relationships may buffer the psychological effects of stress. These findings support the need to move beyond simple risk–outcome models of student mental health. In contexts marked by structural inequality, financial precarity, geographic mobility, and collective expectations of educational success, students’ psychological adjustment is shaped by both stress exposure and the relational resources available to them. Strengthening social support systems within HDIs may therefore be an important component of trauma-informed and contextually responsive student mental health interventions.

## Data Availability

The datasets generated during and/or analyzed during the current study are available from the corresponding author on request.*
